# A Rapid Method for Assaying Thiaminase I Activity in Diverse Biological Samples

**DOI:** 10.1371/journal.pone.0092688

**Published:** 2014-03-27

**Authors:** Clifford E. Kraft, Eric R. L. Gordon, Esther R. Angert

**Affiliations:** 1 Department of Natural Resources, Cornell University, Ithaca, New York, United States of America; 2 Department of Microbiology, Cornell University, Ithaca, New York, United States of America; 3 Department of Entomology, University of California Riverside, Riverside, California, United States of America; University of Florida, United States of America

## Abstract

Vitamin B_1_ (thiamine) deficiencies can lead to neurological disorders, reproductive failure and death in wild and domestic animal populations. In some cases, disease is brought about by the consumption of foods high in thiaminase I activity. Levels of thiaminase activity in these foods are highly variable and the factors leading to production of this enzyme are poorly understood. Here we describe improvements in a spectrophotometric thiaminase I activity assay that measures the disappearance of 4-nitrothiophenol, a favored nucleophile co-substrate that replaces the thiazole portion of thiamine during the inactivation of thiamine by the enzyme. Scalable sample processing protocols and a 96-well microtiter plate format are presented that allow the rapid evaluation of multiple, replicated samples in the course of only a few hours. Observed levels of activity in bacterial culture supernatant, fish, ferns and molluscs using this colorimetric assay were similar to previously published reports that employed a radiometric method. Organisms devoid of thiaminase I, based upon previous work, showed no activity with this assay. In addition, activity was found in a variety of fishes and one fern species from which this enzyme had not previously been reported. Overall, we demonstrate the suitability of this technique for measuring thiaminase I activity within small amounts of tissue and environmental samples with replication levels that were heretofore prohibitive. The assay provides a considerable improvement in the ability to examine and understand the properties of an enzyme that has a substantial influence on organism and ecosystem health.

## Introduction

The important role of thiamine (vitamin B1) in human and animal health has been long-recognized, but a broad appreciation of its role in environmental health has developed more slowly [1]. In animal husbandry, instances of mortality from thiamine deficiency were first described in the 1940s in mink and foxes raised for fur production [2], [3], then later in cattle, sheep and goats [4], [5], [6]. Japanese scientists pursued extensive studies during and following World War II linking thiamine deficiency in humans to thiamine decomposing bacteria [7]. Thiamine deficiencies in wild populations of predatory fish were first recognized in the 1990s as responsible for a widespread mortality syndrome observed for decades in valuable Baltic Sea and Laurentian Great Lakes fisheries [8].

One common dietary link in thiamine-deficiency mortality syndromes observed in commercial fisheries, animal husbandry and humans has been the consumption of food with high thiaminase activity. Two thiamine-degrading enzymes have been described: thiaminase I and thiaminase II or TenA [9]. Thiaminase I catalyzes the replacement of the thiazole moiety of thiamine with any of a variety of organic nucleophiles [10], [11]. Thiaminase II degrades thiamine using a similar mechanism, except water serves as the nucleophile [12], [13]. Thiaminase I is the only thiaminase linked to fish reproductive failure and animal mortality, consequently it has been the focus of studies investigating syndromes associated with enzymatic thiamine degradation [14], [15], [16]. Predatory salmonine fishes suffering from thiamine deficiency in North America and Europe prey upon clupeid fishes with high thiaminase I activity [17], [18]. In addition, feeding experiments showed that mink and foxes died from thiamine deficiency when fed raw fish that contained thiaminase [2], [3]. Finally, domestic animals such as horses and cattle regularly die from thiamine deficiency after feeding on bracken fern that contains high thiaminase I activity [19].

In the 1950s, researchers isolated thiaminase I-producing bacteria from human feces [20], [21]. One of these bacteria was subsequently found in rumen of ailing sheep with cerebrocorticol necrosis, a disease associated with thiamine deficiency [22]. For decades the thiaminase literature has referred to non-microbial sources of thiaminase, such as plants, fish, shellfish (including mollusks), and crustaceans [23], but it is still unclear whether multi-cellular organisms produce a divergent thiaminase I or whether the enzyme activity within these organisms originates from microbial sources [18], [24]. Recent genome sequencing efforts have helped identify additional putative bacterial genes that likely code for thiaminase I homologs while no clear homolog has been identified in sequenced genomes or transcriptomes of fish.

Analytical approaches to assess the thiaminase activity in plant or animal tissues and bacterial cultures have evolved since thiamine-degrading enzymes were described in the 1940s. Sealock *et al.* [25] first used the term “thiaminase” to describe thiamine-degrading enzymes in fish by noting that this substance could be regarded as a type of thiaminase only if its enzymatic nature was confirmed. Shortly thereafter, Krampitz and Woolley [26] reported the distinguishing enzymatic action of a thiaminase (obtained from an aqueous extract of carp viscera) as its ability to split thiamine into its pyrimidine and thiazole moieties, after which Yudkin [27] firmly established use of the term thiaminase in a review of studies of enzymes responsible for paralysis in animals feeding upon fish. It is interesting to note that this early report [26] of an apparent hydrolytic enzymatic cleavage by carp thiaminase of thiamine into its thiazole portion (isolated as 4-methyl-5-hydroxyethylthiazole) and a hydroxylated-pyrimidine portion (isolated as 2-methyl-4-amino-5-hydroxymethylpyrimidine) is characteristic of the type II thiaminase enzyme reaction. Such a finding is in conflict with subsequent studies reported by Fujita [23], Sato et al. [28], Bos and Kozik [29], and Wistbacka et al. [18] in which the action of carp thiaminase enzymes have been consistently characterized as a base-exchange, type I, thiaminase reaction.

Early studies measured thiaminase activity by assaying the disappearance of thiamine in the presence of biological tissues, typically from fish [25], [26], [30]. Several alternative methods were developed [31], [32] before Edwin and Jackman [4] laid the foundation for a radiometric thiaminase assay that was used extensively for the next 40 years (see [18], [33], [34] for subsequent modifications of this method). The radiometric assay uses ^14^C-thiamine as a substrate for thiaminase I, which releases thiazole -2-^14^C that is then extracted and the associated radioactivity is measured. Unfortunately, requirements for handling radioactive materials and the expense and limited availability of radiolabeled thiamine have restricted the use of this assay to a small number of laboratories.

We faced this limitation in conducting studies examining evidence that bacterial symbionts are the source of thiaminase within fishes and other aquatic organisms, which led us to develop a spectrophotometric thiaminase assay [35] to evaluate activity in bacterial cultures, environmental samples and animal tissues. This assay measures the consumption of 4-nitrothiophenol (4-NTP), a favored nucleophile co-substrate that replaces the thiazole portion of thiamine during the inactivation of thiamine by thiaminase I. A preliminary comparison of the radiometric and 4-NTP colorimetric methods was conducted that described refinements in the assay for measuring thiaminase I in samples of fish tissues [36], though this report raised some concern about differences in sensitivity between the two assays. Here we describe further modifications of the 4-NTP assay for high throughput use with a microtiter plate reader, as well as demonstrate its application to a broad variety of organisms previously described as containing thiaminase I. Specifically, we demonstrate the efficacy of the 4-NTP assay to measure thiaminase I in both bacterial cultures and tissues from plants, fish, crustaceans, and molluscs. We also report refinements that improve the ability to quantify high-activity samples and demonstrate that this more accessible assay is as effective as the radiometric assay for biological samples.

## Materials and Methods

### Ethics statement

All fish used in this study were collected in waters of New York State (Table 1) under the authority of a fish collection permit authorized by the Bureau of Fish, Wildlife and Marine Resources of the New York State Department of Environmental Conservation. Alewife, rainbow smelt and hickory shad were collected with gill nets, then were euthanized with MS-222 and frozen according to procedures approved by the Cornell University Institutional Animal Care and Use Committee, which specifically approved this study. All other fishes analyzed in this study were collected using a backpack electrofishing unit, then were euthanized with MS-222 and frozen according to procedures approved by the Cornell University Institutional Animal Care and Use Committee. Quagga mussels were collected from nearshore waters of Cayuga Lake, and no additional permission for collection was required. All ferns analyzed in this study were collected on the private property of one of the authors (ERA), and no additional permission for collection was required. None of these field collections affected endangered or protected species, and GPS coordinates for all sample collection locations are provided in Tables 1 and 2.

**Table 1:** Fish and mussel samples evaluated in this study.

Organism	Common name	Length ± standard deviation	Location collected	Date
*Alosa pseudoharengus*	Alewife	15.2±1.2 cm	43.28 °N 76.33°W	6/12/2010
*Osmerus mordax*	Rainbow smelt	13.1±2.1 cm	43.41°N 74.53°W	5/12/2011
*Alosa mediocris*	Hickory shad	30.5±2.2 cm	39.39°N 76.10°W	4/21/2011
*Fundulus diaphanous*	Banded killifish	4.5±0.7 cm	42.27°N 76.27°W	11/14/2011
*Rhinichthys atratulus*	Blacknose dace	4.8±0.9 cm	42.27°N 76.27°W	11/14/2011
*Campostoma anomalum*	Central stone roller	7.8±0.3 cm	42.27°N 76.27°W	11/14/2011
*Luxilus cornutus*	Common shiner	6.2±0.5 cm	42.27°N 76.27°W	11/14/2011
*Semotilus atromaculatus*	Creek chub	7.6±0.5 cm	42.27°N 76.27°W	11/14/2011
*Exoglossum maxilingua*	Cutlips minnow	7.0±2.3 cm	42.27°N 76.27°W	11/14/2011
*Semotilus corporalis*	Fallfish	15.4±0.1 cm	42.27°N 76.27°W	11/14/2011
*Etheostoma flabellare*	Fantail darter	5.0±1.5 cm	42.27°N 76.27°W	11/14/2011
*Rhinichthys cataractae*	Longnose dace	5.3±0.5 cm	42.27°N 76.27°W	11/14/2011
*Etheostoma olmstedi*	Tesselated darter	6.2±0.8 cm	42.27°N 76.27°W	11/14/2011
*Catostomus commersonii*	White sucker	13.2±3.1 cm	42.27°N 76.27°W	11/14/2011
*Dreissena rostriformis bugensis*	Quagga mussel	1.5 ±0.8 cm	42.35°N 76.37°W	7/4/2011

**Table 2:** Fern samples evaluated in this study.

Organism	Common name	Location collected	Date
*Polystichum acrostichoides*	Christmas fern	42.20 °N 76.08°W	6/29/2011
*Osmunda claytoniana*	Interrupted fern	42.20 °N 76.08°W	6/15/2011
*Dennstaedtia punctilobula*	Hay-scented fern	42.20 °N 76.08°W	6/15/2011
*Onoclea sensibilis*	Sensitive fern	42.20 °N 76.08°W	6/15/2011
*Osmundastrum cinnamomeum*	Cinnamon Fern	42.20 °N 76.08°W	6/16/2011
*Athyrium felix-femina*	Lady fern	42.20 °N 76.08°W	6/16/2011
*Thelypteris noveboracensis*	New York fern	42.20 °N 76.08°W	6/16/2011
*Deparia acrostichoides*	Silvery spleenwort	42.20 °N 76.08°W	7/10/2011
*Dryopteris intermedia*	Evergreen wood fern	42.20 °N 76.08°W	7/10/2011
*Dryopteris carthusiana*	Spinulose fern	42.20 °N 76.08°W	7/10/2011
*Dryopteris marginalis*	Marginal fern	42.20 °N 76.08°W	7/10/2011

### Bacterial strains and growth conditions

*Paenibacillus thiaminolyticus* 8103 [37], *Aneurinibacillus aneurinilyticus* ATCC 12856 and *Bacillus subtilis* PY79 were grown aerobically in a shaking water bath at 37°C in Trypticase Soy Broth (TSB, Difco). *Clostridium sporogenes* ATCC 15579 was grown anaerobically at 37°C in TSB prepared by bubbling with oxygen-free CO_2_ and autoclaving. The medium was transferred into an anaerobic chamber (Coy Laboratory Products) where it was dispensed into sterile glass culture tubes.

### Organism sample collection

Two species of fish common in the Great Lakes region that have high thiaminase I activity, alewife and rainbow smelt, along with twelve other common freshwater fish species of the northeastern U. S. and quagga mussels were collected (Table 1). All fish and mussels were frozen shortly after collection and stored at −80°C until assayed.

Twelve species of ferns were collected from local forests (Table 2). Rhizomes and roots were rinsed in clean well water. Fronds, stipes, rhizomes and roots were separated, placed in freezer bags and stored at −80°C until processed. For all fern species, the rhizomes or fine roots were assayed because these fern structures have the greatest activity in documented thiaminase-positive fern species [19].

Crustacean zooplankton *Daphnia pulicaria* were raised in lab culture and fed chlorophyte algae, *Scenedesmus acutus*, that were also raised in culture. In one treatment these algae were grown in a medium that contained thiamine, and in a contrasting treatment the algae were grown in the same medium without thiamine. Half of the assayed *D. pulicaria* were frozen immediately after collection and stored at −80°C. For the other half of the assayed *D. pulicaria,* the intestinal tract was cleared by placing them in water with clay particles and no algal food for several hours before the zooplankton were collected and frozen in a −80°C freezer.

### Preparing tissues for assay analysis

Large samples (whole fish, plant material) were processed by cutting frozen tissue into small pieces (∼1 cm^3^) with kitchen shears and then placing these into a coffee bean grinder (Hamilton Beach) with a similar amount of dry ice pellets. Samples were not allowed to thaw before being ground to a coarse powder and transferred to a freezer bag. The bag was left open and placed in a −20°C freezer overnight or left at room temperature for a short time to allow the dry ice to sublimate. Once the sample was free of dry ice, as determined by the loss of the white texture of the sample and by the presence of fluid droplets, approximately 1 g of powdered sample was placed in a centrifuge tube. The sample was weighed and suspended in 2.5 times (weight-to-volume) of 100 mM phosphate buffer, pH 6.5. After vortexing, and allowing large pieces to settle, the supernatant was transferred to a 1.5 ml microcentrifuge tube and centrifuged at 16,000×*g* for 10 min. Cloudy supernatant was transferred to a Pierce centrifuge column (89868) and centrifuged again. Generally 3 μl of sample supernatant was used in each assay well.

Smaller samples (fish muscle or gill tissue, quagga mussels removed from the shell) were homogenized in buffer using a Tissuemiser (Fisher Scientific), then the homogenate fluid was clarified by two rounds of centrifugation, as described above. Alewife intestine samples were analyzed intact with their internal contents. Small and soft tissue samples (small mussels) were placed in a 1.5 ml microcentrifuge tube with 2.5 times volume of phosphate buffer and ground with a pestle (USA Scientific), then this mixture was clarified with centrifugation as above. Note that for small samples with low activity, we would occasionally repeat the extraction using a smaller amount of buffer (generally 1 times the volume of phosphate buffer was added to a tissue sample) and rerun the assay. Small samples that were not easily homogenized with a plastic pestle (e.g. zooplankton) were placed in a 2 ml screw cap tube with an equal volume of zirconia/silica beads (BioSpec Products), then a small amount of buffer was added and the mixture was processed with a mini-beadbeater (BioSpec) for 3 minutes. Tubes were centrifuged as above.

Culture supernatant was used in bacterial thiaminase activity assays. A 0.5 ml aliquot of the culture was centrifuged at 16,000×*g* for 1 min. Supernatant was transferred to a clean tube and stored at −80°C until assayed. Generally, 3 μl of supernatant was used in each assay well. The lower detection limit of the thiaminase assay was evaluated by testing nine 1∶1 serial dilutions (with buffer) from the frozen supernatant of a one-day-old *P. thiaminolyticus* culture.

### 4-NTP thiaminase I assay

This assay relies upon measuring the disappearance of the yellow co-substrate 4-NTP, which is used by thiaminase I to inactivate thiamine by replacing the thiazole portion of thiamine with a favored nucleophile co-substrate [35]. All chemicals were purchased from Sigma-Aldrich except Tris(2-carboxyethyl)phosphine hydrochloride (TCEP), which was purchased from Soltec Ventures. Reagents were prepared as described previously with modifications [36]. TCEP buffer (100 mM NaCl; 50 mM phosphate buffer, pH 6.9; 10 mM TCEP) was placed on ice and bubbled for 15 min with O_2_-free N_2_ (which was passed over heated copper filings to remove residual O_2_ [38]) then used immediately. A stock solution of 4-nitrothiophenol (4-NTP), 3 mg/ml dissolved in dimethyl sulfoxide, was diluted to a final concentration of 200 μM 4-NTP in TCEP buffer or TCEP buffer plus 400 μM thiamine HCl. The 4-NTP-TCEP solution with thiamine is referred to as the “experimental” solution and the solution with no added thiamine is the “control”.

A 96-well microtiter plate reader was used to assay the disappearance of 4-NTP, using either a 12-sample or 24-sample format. The 12-sample format provided 4 replicates of experimental and control wells from a tissue extract or culture type; the 24-sample format provided duplicates of each. All wells of the microtiter plate were filled with 97 μl of solution using a multi-channel pipette; half of these wells being filled with the experimental solution and half with the control. Each well also received 3 μl of sample extract or culture supernatant before being placed in Powerwave XS2 plate reader (BioTek) that was preheated and maintained at 37°C. Readings of absorbance at 411 nm were taken every minute over the course of 1 hour to record the disappearance of the yellow 4-NTP. A path-length correction feature of the plate reader was used to standardize absorbance to a 1 cm path length.

### Data analysis

The plate reader Gen5 software (BioTek) was used to determine the maximum velocity of 4-NTP degradation by comparing rates of change in optical density over all of the possible 10-min intervals. The maximum values were averaged for replicate wells. To correct for non-enzymatic degradation of 4-NTP, the mean maximum velocity of the control wells (without thiamine) for each sample was subtracted from the mean maximum velocity of the experimental wells. Change in absorbance was converted into a rate of 4-NTP degradation using its extinction coefficient in TCEP: 13,650 M^−1^·cm^−1^ [35]. The 4-NTP cosubstrate concentration (moles/L) was converted to moles by multiplying by the reaction volume (10^−4^ L). For samples with a low raw activity (≤ 25 pmol 4-NTP degraded·min^−1^) but greater than a minimum detectable raw value (4.8 pmol 4-NTP degraded·min^−1^), the significance of observed differences in the maximum velocities of control wells and experimental wells was evaluated using an unpaired *t*-test. If a significant difference was found (*P*-value < 0.05), the presence of thiaminase I activity was considered to have been substantiated. 4-NTP degradation rates were converted to thiaminase activity, assuming a 1∶1 degradation of thiamine:4-NTP. For all calculations, we used 1 ml  =  1 g. Measures for tissue extracts were converted to mass-specific rates of thiamine degradation (nmol thiamine degraded·min^−1^·g^−1^) as follows. The raw thiaminase activity value was divided by the combined weight of the buffer and the sample multiplied by the amount of prepared sample added to the assay. Typically, the raw value was converted by multiplying it by the factor ([1 g/3.5 g] · 0.003 g)^−1^. For bacterial culture supernatant assays, the measured activity was standardized to activity per ml of culture, and converted using the factor (0.003 ml)^−1^.

## Results

**Figure 1:**
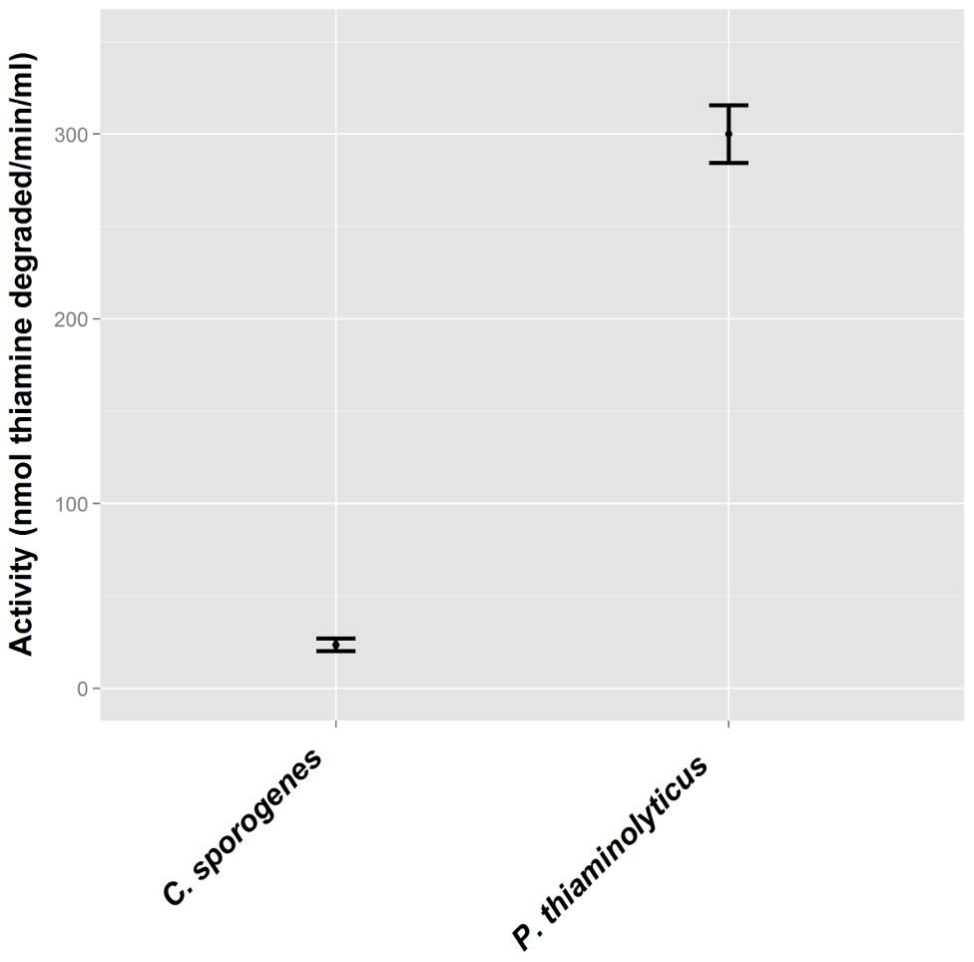
4-NTP assay detection of thiaminase I activity in bacteria known to produce this thiamine-degrading enzyme. Cell-free supernatant from replicate 1-day old cultures of *C. sporogenes* and *P. thiaminolyticus* were tested for thiaminase activity using the 4-NTP assay. Error bars represent the standard error of three replicates.

In agreement with previous reports [38], high thiaminase I activity was found in the supernatant of one-day-old cultures of *C. sporogenes* and *P. thiaminolyticus*, with greater activity observed in cultures of *P. thiaminolyticus* (Figure 1). No thiaminase I activity was detected with the 4-NTP assay in culture supernatants or cell lysates of *A. aneurinilyticus* or *B. subtilis*, bacteria with thiaminase II activity [12], [23].

**Figure 2:**
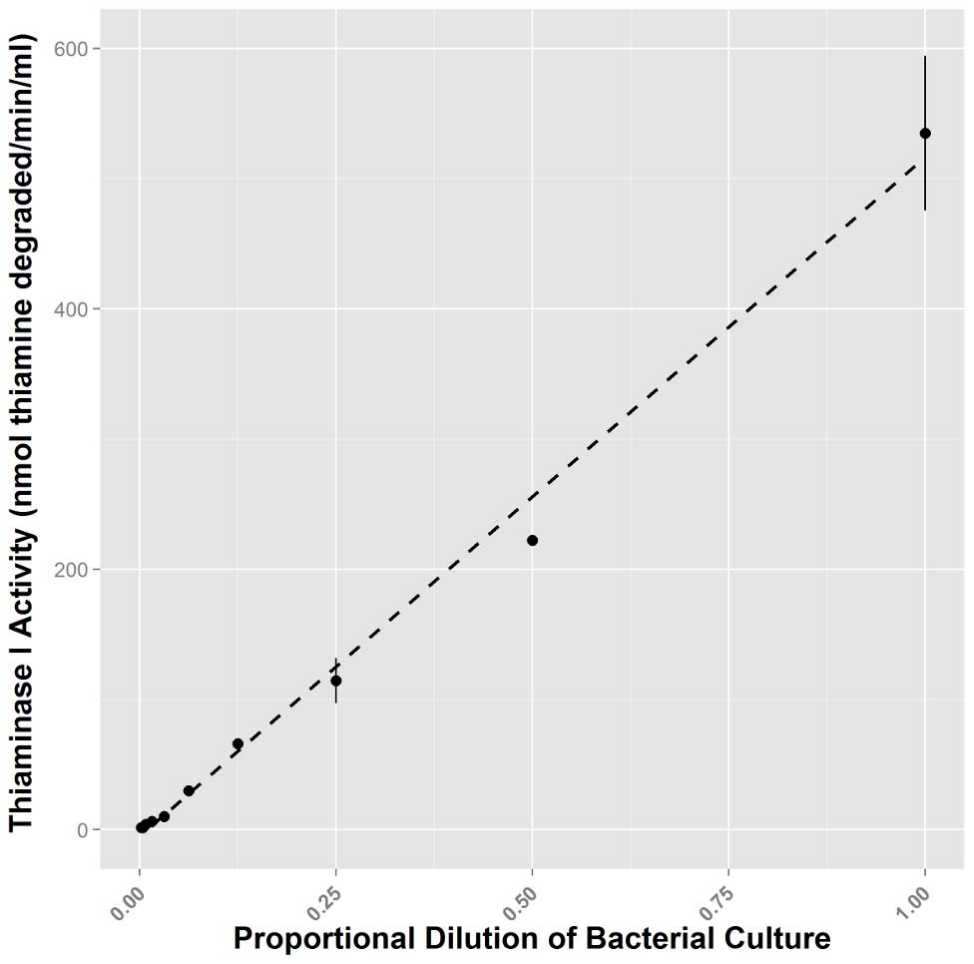
Assessing 4-NTP assay sensitivity with a dilution series. Culture supernatant from *P. thiaminolyticus* was serially diluted 1∶1 with buffer and each dilution assayed. Thiaminase activity was detected over a large range of activities from 534 nmol thiamine degraded·min^−1^·ml^−1^ to approximately 1.6 nmol degraded·min^−1^·ml^−1^. Error bars represent the standard error of 3 replicates.

The dilution series of *P. thiaminolyticus* supernatant exhibited a proportional decline in thiaminase I activity down to a dilution level of 1/256 (Figure 2). This represents a raw activity value of approximately 4.8 pmol thiamine degraded·min^−1^ or an equivalent activity of about 1.6 nmol thiamine degraded·min^−1^·ml^−1^. We therefore consider this assay to have sensitivity down to that value (i.e. below which the assay failed to distinguish a further decrease in thiaminase I activity). Measured values below that level were considered to have an activity of zero. The difference in absorbance at 411 nm between control and experimental wells for a sample with the minimum detectable raw activity value of 4.8 pmol 4-NTP degraded • min-1 was 6.6 mOD units.

**Figure 3:**
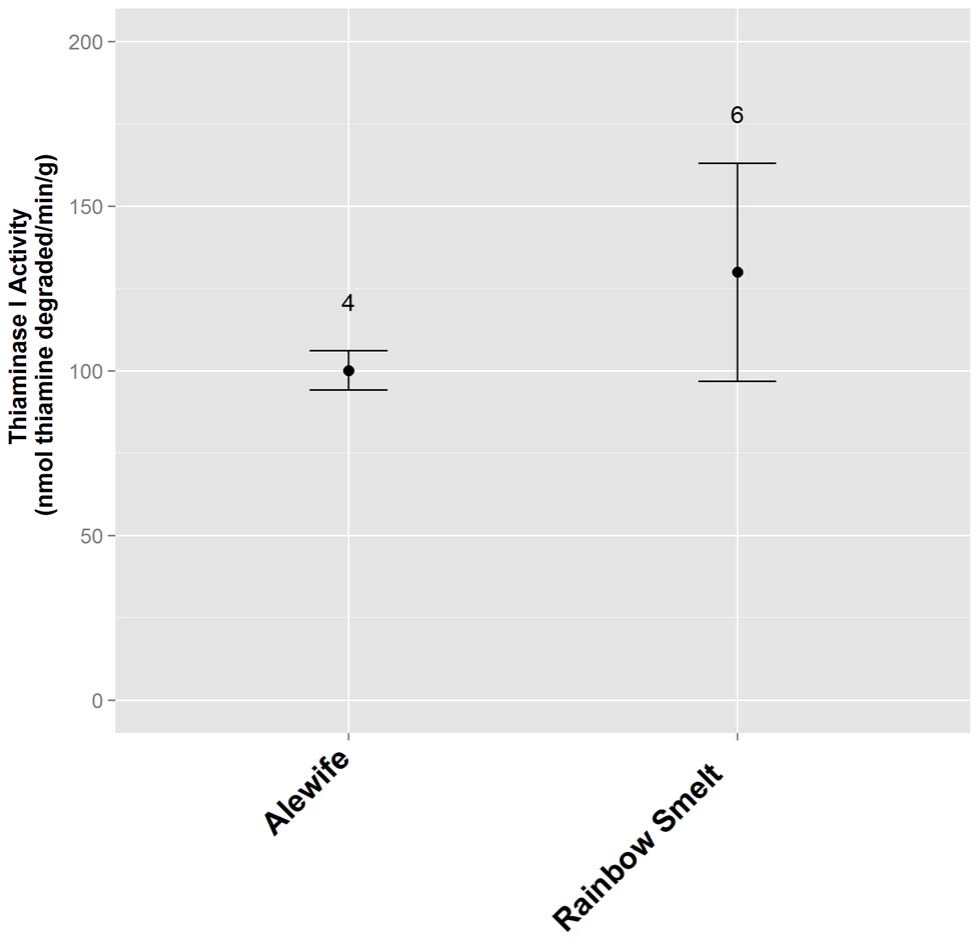
4-NTP assay results for Laurentian Great Lakes fishes commonly found to have thiaminase I activity. Aqueous extracts from whole ground fish were assayed. The number of individual fish tested is shown above each assay value, and error bars represent the standard error of replicates.

**Figure 4:**
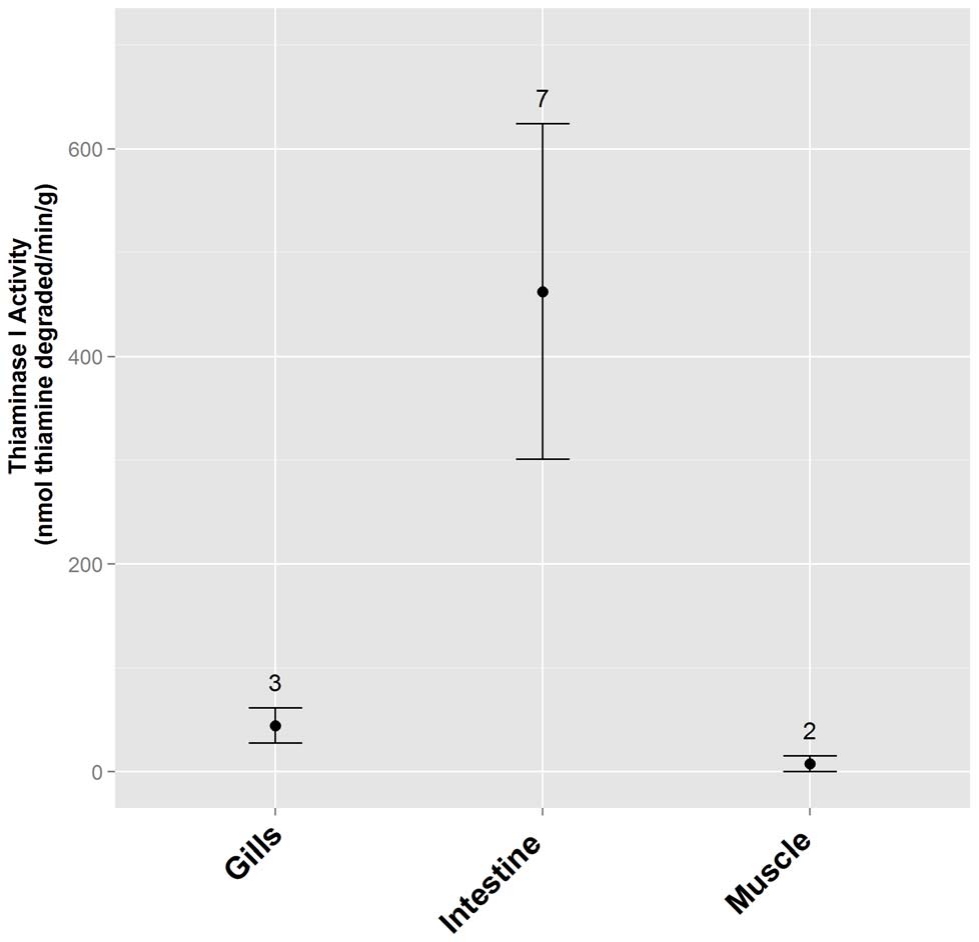
4-NTP assay results from tissues within a dissected alewife. The number of individual fish tested is shown above each assay value, and error bars represent the standard error of replicates. Largest thiaminase I activity values were associated with the intestine and its contents.

**Figure 5:**
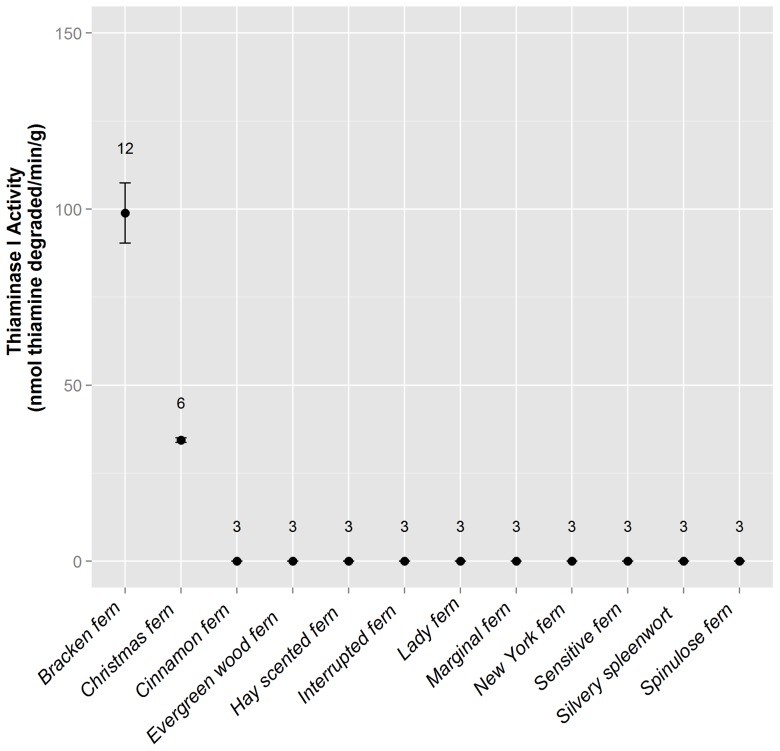
Thiaminase I activity in New York woodland ferns. Replicate samples (number of replicates shown above the plotted activity value) from rhizomes or fine roots of ferns were assayed. Bracken fern thiaminase I activity was similar to previously published reports. Thiaminase I activity was detected in only one additional fern species (Christmas fern). Error bars represent the standard error of replicates.

Substantial thiaminase I activity was found in whole alewife and rainbow smelt (Figure 3), with greater levels of activity localized to specific tissues within alewife, most notably the intestine (Figure 4). High levels of thiaminase I activity were found in bracken fern rhizomes, and lower levels of activity were found in Christmas fern rhizomes and fine roots, but no activity was detected in rhizomes or fine roots from ten other ferns common to upstate New York (Figure 5). The greatest activity was found in the rhizomes or roots of bracken or Christmas ferns, by comparison with the stipe and fronds of the same plant (bracken fern stipe and fronds: 0 and 29 nmol thiamine degraded·min^−1^·g^−1^, respectively; Christmas fern stipe and fronds: no activity was found).

**Figure 6:**
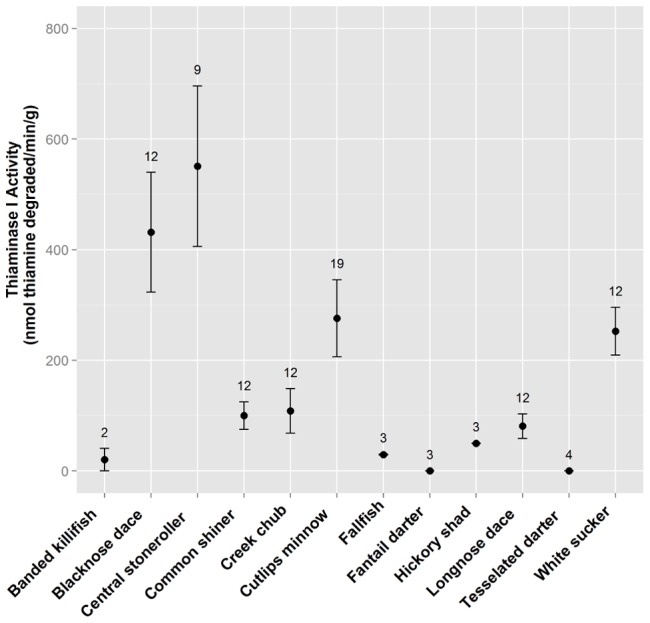
Thiaminase I activity in north temperate stream fishes. Twelve common (to New York) fish species were tested for thiaminase activity and all but two tested positive. Number of individuals (2 to 19) assayed is shown above the plotted activity value. Error bars represent the standard error of replicates.

Thiaminase I activity was detected in 9 of 11 species of fish collected from a tributary to Cayuga Lake (Fall Creek) and one species collected from the Susquehanna River (Figure 6). Thiaminase I activity of Cayuga Lake quagga mussels was 60 nmol thiamine degraded·min^−1^·g^−1^ (± se 22). No thiaminase I activity was found in 14 samples of *D. pulicaria* that were assayed.

## Discussion

Thiaminase I activity levels determined by the 4-NTP assay were consistent with previous observations of activity in bacteria and fish, ferns, molluscs, and specific tissues within these organisms. A key comparison showed that high levels of thiaminase I were found in cultures of two bacteria from which this enzyme was first identified in the 1950s: *C. sporogenes* (renamed from *Clostridium thiaminolyticum*) and *P. thiaminolyticus* [16], [21]. Another important comparison showed that substantial thiaminase I activity was found in two species of Laurentian Great Lakes forage fish, alewife and smelt, regularly reported to contain this enzyme. For example, Tillitt *et al.* [39] reported mean levels of thiaminase I activity in Lake Michigan alewife and rainbow smelt as 4.4 and 2.6 nmol·g^−1^·min^−1^, respectively, by comparison with our mean values of 100 nmol·g^−1^·min^−1^ for alewife collected from Lake Ontario and 129 nmol·g^−1^·min^−1^ for smelt from an Adirondack lake. However, these are general comparisons from fish captured in different circumstances, and substantial variability has been evident in previously published estimates of thiaminase I activity in different individual fishes [39]. The 4-NTP assay verified much greater activity in alewife intestinal tissues (462 nmol·g^−1^·min^−1^) when compared with gill (45 nmol·g^−1^·min^−1^) and muscle (7 nmol·g^−1^·min^−1^) tissue; these values are greater than the only previous report of thiaminase I activity in intestinal tissue from a single Lake Michigan alewife (33 nmol·g^−1^·min^−1^; [34]).

4-NTP assay results in this study were consistent with numerous observations of high thiaminase activity in bracken fern and specific tissues within these plants, with the enzyme most abundant in the rhizomes compared to the stipe and fronds [19]. Evans reported thiaminase I levels in bracken fern rhizomes ranging from approximately 80 to 480 nmol·g^−1^·min^−1^ (estimated from Figure 1 in [19]), comparable to our mean of 126 nmol·g^−1^·min^−1^ in rhizomes. Furthermore, we found one additional species of fern (*P. acrostichoides*) with thiaminase I activity that belongs to the same genus as a British fern (*Polystichum setiferum*) previously reported to contain thiaminase I [40]. Our assay measurements resulted in a mean value of 34 nmol·g^−1^·min^−1^ in rhizomes from *P. acrostichoides*, by comparison with reported activity in *P. setiferum* ranging from 8 to 16 nmol·g^−1^·min^−1^ [40]. Activity in quagga mussels assessed with the 4-NTP assay was within the range reported by Tillitt *et al.* [41]: 19.5 to 223.8 nmol·g^−1^·min^−1^.

In contrast to previous assays, the 4-NTP assay as described here provides the ability to analyze large numbers of samples at a minimal expense, without compromising detection of biologically relevant levels of thiaminase activity. Notably, the materials, equipment, technician time and training required to run the assay – as well as the ability to conduct the assay in a lab that does not require special permits or procedures for using radio-labeled thiamine – are minimal by comparison with the radiometric assay. A 96-well microplate reader provides the capacity for 12, 16 or 24 samples allowing for 4, 3 or 2 replicates, simultaneously. In previous experiments, the use of the radiometric assay restricted the number of samples that we could analyze given the fixed budget constraints of a research grant [42]. We also suspect that expense constrained the use of replicates in thiaminase studies conducted decades ago [19], [40]. The limitations of running the radiometric assay also seemed apparent in recent studies, such as an analysis of the distribution of thiaminase I in alewife tissues conducted using a single fish [34] or an analysis of thiaminase I in dreissenid mussels that was restricted to three samples for each collection event [41].

We consider it important to highlight several choices made in calculating enzyme activity from our assay procedure. First, in comparing activity measures from the 4-NTP assay within biological tissues to previously reported values determined using different assays, we considered our samples to have been assayed in units of wet weight instead of dry weight because these samples were not dried during sample preparation. By contrast, thiaminase I activity levels determined using a radiometric method applied to ferns have been reported in units of dry weight [19]. Second, for bacterial cultures we chose to report enzyme activity values in units that are not mass-specific (nmol thiamine degraded·min^−1^·ml^−1^) because no consistent meaningful mass-specific unit conversion could be readily determined for bacterial culture supernatant. We considered using total protein for this conversion but reasoned that because thiaminase activity increases in culture supernatants over the course of 3 days (data not shown) that the concentration of secreted thiaminase I relative to total protein in the supernatant would vary with culture age in an unpredictable manner.

Use of a microtiter plate reader to take spectrophotometric measurements over a 60-min period facilitated determining the maximum activity (slope) of the thiaminase I enzyme within a given sample. We chose a 10-min window over which to determine maximum enzyme activity from trial assessments of activity in bacterial culture samples with different levels of activity. The greatest rates were typically seen early in the hour-long time course and exceptions to this only occurred when the rate was steady throughout the measurement period. We also note that samples with high thiaminase activity required dilution to ensure that sufficient 4-NTP remained throughout the duration of the assay.

We do not suggest a minimum biological sample size at which the 4-NTP assay can be used because detectable activity would depend in part on the level of activity in the material. However, the smallest amount of tissue from which we detected activity was 10 mg from quagga mussels. By comparison, Zajicek *et al.* [34] reported typically analyzing 1.0 g samples when using the radiometric assay, but smaller samples (50.0–250.0 mg) were also assayed. In that study, a 50 mg pellet of lyophilized *P. thiaminolyticus* was processed and activity measured in subsequent dilutions, though they did not use these results to evaluate the detection limit of the radiometric assay.

Our fish and fern tissue analysis results are consistent with the hypothesis that microbial sources are responsible for the presence of thiaminase I in fishes, as well as other studies of the distribution of this enzyme in a variety of fishes. Early work by Fujita [43] reported high thiaminase activity in specific fish organs, including the spleen and kidney of gray mullet (*Mugil cephalus*), the liver and gills of yellowfin goby (*Acanthogobius flavimanus*), and the kidney, intestine, gills, ovaries and liver of carp (both *Carrassius auratus* and *Cyprinus carpio*). In making these observations Fujita used the disappearance of thiamine as a measure of “thiaminase” activity, as did several subsequent studies that measured thiamine-degrading activity and reported it as thiaminase I activity. For example, Sato *et al.* [28] reported thiaminase I as concentrated in the kidney and spleen of carp (*Cyprinus carpio*), and Arsan and Malyarevskaya [44] reported high activity in the liver and intestines of silver carp (*Hypophthalmichthys molitrix*). Some of these tissues (e.g. gastrointestinal tracts) contain abundant bacteria, and it is notable that we found very low levels of thiaminase in muscle tissue where bacteria are unlikely to occur. A similar pattern was reported by Fujita [43] in an overview of thiaminase in 17 shellfish species and seven crustaceans, in which muscle levels of thiaminase were zero or barely detectable when tested separately from viscera or other tissues from individuals that had high “total organism” levels of thiaminase. The greater levels of thiaminase I observed in bracken fern rhizomes provide similar evidence in support of the hypothesis that bacteria are the source of thiaminase I in these plants, given observations of the commonly observed localized distribution of bacteria in plant roots by contrast with other tissues [45].

We have demonstrated the availability of a high throughput assay that can rapidly test for thiaminase I activity in bacterial cultures, environmental samples and animal tissues. We expect that use of this assay will provide a valuable method to further foster an understanding of the important role of thiaminase I in nature by facilitating the application of controlled and replicated experiments as well as fine-scale analysis of environmental samples.
